# Virginia State Dental Association

**Published:** 1886-09

**Authors:** 


### Virginia State Dental Association.

It was my privilege to be present at the annual meeting of the Virginia State Dental Association, held at Natural Bridge on the 10th of August and the three succeeding days.

It is one of our most energetic working State Societies, and it includes most of the best men of the profession in Virginia. This meeting was one of the largest ever held, and one of the most productive as far as its work was concerned.

Excellent papers were read, and interesting discussions had upon them. We have never met a more cordial and genial body of men, thoroughly interested in the work on hand.

It was a real pleasure to meet such a man as Dr. W. W. H. Thackston, of whom the whole profession may well be proud; the oldest living graduate of a Dental College in the world, and others standing with him worthy of high regard, such as Dr. J. H. Moore, Dr. Turner, of North Carolina, Dr. Hodgkin, of Washington City, and many others.

This Society has been instrumental of great good for the profession in Virginia, and we doubt not, that much more fruit will be borne by it in the future.

Would that every member of the profession in each State of our Union felt the great interest in their respective State Societies that is exhibited in Virginia.

